# The Sounds of the Heart

**Published:** 1842-05

**Authors:** M. Cruveilhier


*On the Sounds of the Heart.* By M. Cruveilhier.—From his recent investigations on this subject, M. Cruveilher is led to infer, contrary to the general belief, and certainly in opposition to our own researches, that both sounds of the heart are seated at the origin of the pulmonary artery and the aorta, and that they are owing to the clacking of the semilunar valves:—that the first sound which coincides with the systole of the ventricles, and the dilatation of the arteries, is the result of the elevation of the semilunar valves which had been previously depressed,—and that the second, which coincides with the dilatation of the ventricles and the contraction of the arteries, is owing to the depression of the semilunar valves by the refluent blood.

The subject of the sounds of the heart is ably discussed in the work of Dr. Hope, commenced in this number of the "Library," which contains, in addition to the experiments of European observers, those of the able and estimable American Editor, Dr. Pennock.—*Am. Med. Library and Intell.*

